# An Engineered Outer Membrane-Defective *Escherichia coli* Secreting Protective Antigens against *Streptococcus suis* via the Twin-Arginine Translocation Pathway as a Vaccine

**DOI:** 10.4014/jmb.2107.07052

**Published:** 2022-02-11

**Authors:** Wenyu Li, Fan Yin, Zixuan Bu, Yuying Liu, Yongqing Zhang, Xiabing Chen, Shaowen Li, Lu Li, Rui Zhou, Qi Huang

**Affiliations:** 1 State Key Laboratory of Agricultural Microbiology, College of Veterinary Medicine, Huazhong Agricultural University, Wuhan 430070, P.R. China; 2 Cooperative Innovation Center for Sustainable Pig Production, Wuhan 430070, P.R. China; 3 International Research Center for Animal Disease, Ministry of Science and Technology, Wuhan 430070, P.R. China; 4 Key Laboratory of Development of Veterinary Diagnostic Products, Ministry of Agriculture and Rural Affairs of China, Wuhan 430070, P.R. China; 5 Institute of Animal Husbandry and Veterinary Science, Wuhan Academy of Agricultural Science and Technology, Wuhan 430070, P.R. China; 6 Shandong Vocational Animal Science and Veterinary College, Weifang, P.R. China

**Keywords:** Twin-arginine translation (Tat) system, *Escherichia coli*, *Streptococcus suis*, antigen secretion, live vector vaccine, immunoprotection

## Abstract

Live bacterial vector vaccines are one of the most promising vaccine types and have the advantages of low cost, flexibility, and good safety. Meanwhile, protein secretion systems have been reported as useful tools to facilitate the release of heterologous antigen proteins from bacterial vectors. The twin-arginine translocation (Tat) system is an important protein export system that transports fully folded proteins in a signal peptide-dependent manner. In this study, we constructed a live vector vaccine using an engineered commensal *Escherichia coli* strain in which *amiA* and *amiC* genes were deleted, resulting in a leaky outer membrane that allows the release of periplasmic proteins to the extracellular environment. The protective antigen proteins SLY, enolase, and Sbp against *Streptococcus suis* were targeted to the Tat pathway by fusing a Tat signal peptide. Our results showed that by exploiting the Tat pathway and the outer membrane-defective *E. coli* strain, the antigen proteins were successfully secreted. The strains secreting the antigen proteins were used to vaccinate mice. After *S. suis* challenge, the vaccinated group showed significantly higher survival and milder clinical symptoms compared with the vector group. Further analysis showed that the mice in the vaccinated group had lower burdens of bacteria load and slighter pathological changes. Our study reports a novel live bacterial vector vaccine that uses the Tat system and provides a new alternative for developing *S. suis* vaccine.

## Introduction

Non-pathogenic or virulence-attenuated bacteria, such as *Escherichia coli*, *Lactobacillus*, *Bacillus*, *Salmonella*, and *Listeria*, when genetically engineered to secrete heterologous proteins, can be exploited as tools for antigen delivery [1]. Among the prominent advantages of live bacterial vaccines are low cost, flexible vaccination routes, and enhanced induction of immune response [1-3]. *E. coli* is the most widely used “workhorse” due to its high simplicity in genetic manipulations and high efficiency [4-6]. However, as a gram-negative bacterium, the secretion of heterologous proteins from the cytoplasm to the extracellular environment is challenging due to the presence of an outer membrane. One of the strategies that have been applied for heterologous protein secretion by *E. coli* is the utilization of bacterial protein secretion systems [7, 8]. So far, several bacterial protein secretion systems have been devised to facilitate heterologous proteins to be anchored to the cytoplasmic membrane [9], translocated to the periplasm [10, 11], displayed on the bacterial surface [12], secreted to the extracellular environment [13], or even injected into the eukaryotic cells [14]. By employing these secretion systems, several live bacterial vector vaccines have been developed. By using an attenuated enteropathogenic *E. coli* (EPEC) as a live vaccine vector, secretion of Shiga toxin B subunit was achieved via an *E. coli* hemolysin secretion (type I secretion) apparatus, and the rabbits inoculated with the recombinant live vaccine showed partial protection from infection of a virulent EPEC strain [15]. *Staphylococcus aureus* antigens SaEsxA and SaEsxB were successfully delivered into the cytosol of host cells when incorporated into the *Salmonella* type III secretion system, which elicited both humoral and cellular immune response, providing protection from *S. aureus* infection [16].

The twin-arginine translocation (Tat) system is a protein export system that recognizes its cargo proteins through the Tat signal peptide featured with an S/T-R-R-x-F-L-K motif, and exports them to the periplasm, or inserts them into the membrane [11, 17]. A distinct feature of the Tat system is that it allows the folded protein to be exported. The Tat system has been shown to have the potential for exporting therapeutic and industrial proteins, including growth hormones, interferon, single-chain fragment variable (scFV) [18, 19]. Recently, Albiniak *et al*. reported a high-level secretion of GFP using an *E. coli* strain in which its native Tat system was replaced with a *Bacillus* Tat system [20]. Moreover, the Tat system has been used to construct a multivalent vaccine [21].

*Streptococcus suis* is a zoonotic bacterial pathogen that causes huge economic loss while also posing a threat to human health [22, 23]. As carriage of *S. suis* is prevalent in pig herds and antimicrobial resistance is severe, controlling *S. suis* infection will still hinge on the use of vaccines. Both bacterins and subunit vaccines have been developed [24]. However, due to the complexity of *S. suis* epidemiology in swine, there is still a lack of universal vaccines providing cross-protection and effective control of *S. suis* infection. Therefore, developing novel vaccines is critical.

In this study, we show that protective antigen proteins of *S. suis*, including SLY, Enolase, and Sbp, when fused with a Tat signal peptide, can be secreted to the extracellular environment by using *E. coli* MC4100 Δ*amiA*Δ*amiC*, a commensal *E. coli* strain with compromised outer membrane integrity. Using the antigen-secreting *E. coli* strains to orally inoculate mice, we show that the vaccinated mice displayed milder clinical symptoms in the lung, brain, and spleen, lower bacteria load, and a higher survival rate after *S. suis* challenge.

## Materials and Methods

### Bacterial Strains and Plasmids

Bacterial strains and plasmids used in this study are listed in Table 1. The DNA sequence of primers was listed in Table S1. *E. coli* was normally grown in lysogeny broth (LB) or on LB agar at 37°C. When necessary, chloramphenicol, ampicillin, and spectinomycin were used at a final concentration of 25 μg/ml, 100 μg/ml, and 100 μg/ml, respectively. *E. coli* MC4100 Δ*tatABC* and *E. coli* Δ*amiA*Δ*amiC* strains were constructed using CRISPR/Cas9 technology as previously described with some modifications [25]. Briefly, the pREDCas9 plasmid was used to transform *E. coli* MC4100 competent cells. The pREDCas9-containing *E. coli* strain was cultured until the absorbance at OD_600_ reached 0.3 in LB containing 0.1 mM IPTG to induce the expression of the λ RED recombination system, and then competent cells were prepared. A mixture containing the pgRNA-bacteria plasmid expressing sgRNA targeting the gene of interest and a DNA fragment containing the 500 bp upstream and the 500 bp downstream regions of the target gene was used for transformation. The colonies were selected and PCR was performed to test the presence of the target gene. The pgRNA was then eliminated by culturing the cells in the presence of 0.2% (w/v) arabinose at 30°C, and the pREDCas9 plasmid was cured by growing the cells at 42°C.

Plasmids were constructed by seamless cloning using the ClonExpress MultiS One Step Cloning Kit (Cat. No. C113, Vazyme Biotech Co., Ltd., China). pQE80-SufIss-GFPhis was constructed by ligating the EcoRI- and HindIII-digested pQE80, the SufI signal peptide-encoding DNA, and a DNA fragment containing the GFP-encoding sequence by seamless cloning. pJ23 was constructed by replacing the T5 promoter and lac operator with the constitutive promoter J23119 (Part: BBa_J23119). pJ23-SufIss-SLYhis plasmid was constructed by ligating the EcoRI and HindIII-digested pJ23, the SufI signal peptide-encoding DNA, and a DNA fragment containing the signal peptide-less SLY-encoding sequence with His tag by seamless cloning. pJ23-SufIss-Enohis and pJ23-SufIss-Sbphis were constructed in the same way. pgRNA-*tatABC* was constructed by replacing the fragment between AatII and SalI sites of pgRNA-bacteria with the fragment expressing the crRNA targeting *tatABC*. pgRNA-*amiA* and pgRNA-*amiC* were constructed in the same way.

### Protein Methods

Subcellular fractionation was carried out as described previously [29]. Briefly, overnight cultures of bacteria were subcultured 1:100 to 100 ml LB and incubated at 37°C with shaking until the absorbance at OD_600_ reached 1. To prepare whole-cell samples, 5 ml of culture was pelleted and the cells were resuspended with 250 μl of resuspension buffer (50 mM Tris-HCl, pH 7.6, 2 mM EDTA) followed by ultrasonic fragmentation for 10 min. To prepare periplasmic samples, cells were pelleted from 25 ml of culture and resuspended with 500 μl of fractionation buffer (20 mM Tris-HCl, pH 7.6, 2 mM EDTA, 20% sucrose). Then, lysozyme (final concentration 0.6 mg/ml) was added, and the supernatant was collected after 20 min incubation at room temperature, which was the periplasmic sample. The prepared whole-cell samples and the periplasmic fraction samples were subjected to Western blotting. To test the secretion of antigen proteins to the culture medium, cells of *E. coli* MC4100 strain or Δ*amiA*Δ*amiC* strain expressing the indicated SufI signal peptide-fused antigen proteins were grown to the mid-log phase. Then, the same amount of culture supernatant was taken and concentrated by ultrafiltration and the same amount of sample was analyzed by Western blotting. RNA polymerase was used as the control.

### Immunization and Challenge

All animal experiments were approved by the Laboratory Animal Monitoring Committee of Huazhong Agricultural University and performed according to the recommendations in the Guide for the Care and Use of Laboratory Animals of Hubei Province, China (Approval No. HZAUMO-2020-0070). Female Kunming mice were purchased from the Experimental Animal Center, Huazhong Agricultural University, Wuhan, China. Three strains containing plasmid pJ23-SufIss-SLYhis, pJ23-SufIss-Enohis, and pJ23-SufIss-Sbphis, respectively, were cultured to the mid-log phase at 37°C with shaking. The cells of each strain were mixed at a 1:1:1 ratio, pelleted, and washed three times with sterile saline. Then, 4- to 5-week-old Kunming mice were used for vaccination in which 100 μl of the bacteria mixture containing 10^10^ CFU cells was administered by oral gavage. A vector group was set in parallel which was administered with100 μl of the bacterial mixture containing 10^10^ CFU of *E. coli* Δ*amiA*Δ*amiC* cells. The mice were vaccinated again after two weeks. Seven days after secondary immunization, both groups of mice were injected intraperitoneally with 1.5 × 10^9^ *S. suis* SC19 strain. Clinical symptoms were recorded. At 24 h post-infection, 4 mice were euthanized, and the brain, spleen, and lung were collected, homogenized, diluted, and plated on 5% bovine fetal serum-containing TSA plates for bacteria enumeration.

### Histopathological Examination

The brain, spleen, and lung tissues of the mice in each group were collected, fixed in 4% paraformaldehyde, and embedded in paraffin. The samples were cut into thin sections, stained with haematoxylin/eosin (H&E), and examined under a light microscope.

## Results

### Secretion of Tat Signal Peptide-Fused GFP to the Extracellular Milieus Is Enhanced in the Outer Membrane-Defective *E. coli* Strain

**Fig. 1:**
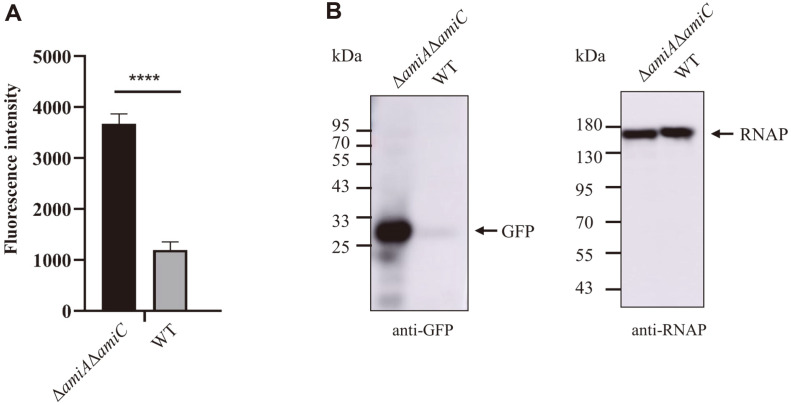
Secretion of SufI signal peptide-fused GFP. Cells of *E. coli* MC4100 (WT) or *E. coli* MC4100 Δ*amiA*Δ*amiC* strain containing pQE80-SufIss-GFPhis were grown overnight at 37°C in the presence of 1 mM IPTG. The culture was centrifuged and the supernatant was harvested. The green fluorescence intensity was measured by using a plate reader (**A**). The supernatant was concentrated by ultrafiltration and the sample was analyzed by Western blotting using anti-GFP antibody and anti-RNA polymerase (RNAP) antibody, respectively (**B**).

The Tat system can transport correctly folded proteins into the periplasm by recognizing the amino-terminal signal peptide of the substrate proteins [11]. Due to the presence of the outer membrane, it is normally difficult for the periplasmic proteins to be further secreted to the extracellular environment. However, it has been shown that the deletion of *amiA* and *amiC*, which encode two Tat-dependent amidases, compromises the integrity of the outer membrane [30]. This enlightened us to test whether proteins exported to the periplasm via the Tat system could be further secreted to the extracellular environment by such an outer membrane-defective strain. To test this, SufIss-GFP, a fusion containing SufI signal peptide and green fluorescent protein, which has been shown as able to be exported by the Tat system, was expressed from pQE80-SufIssGFPhis plasmid in *E. coli* MC4100 (WT) and *E. coli* MC4100 Δ*amiA*Δ*amiC* strains, respectively. By collecting the culture supernatant and measuring green fluorescence intensity, it was shown in Fig. 1A that the green fluorescence intensity in the supernatant of the Δ*amiA*Δ*amiC* strain was significantly higher than that of the WT strain. Western blot results also showed that much more GFP was present in the culture supernatant of the Δ*amiA*Δ*amiC* strain than the WT strain (Fig. 1B), while a comparable amount of RNA polymerase was detected in the culture supernatant for the two strains (Fig. 1C). These results suggest that the Tat signal peptide-fused protein could be secreted to the extracellular environment by the outer membrane-defective *E. coli* strain.

### The SufI Signal Peptide-Fused *S. suis* Antigen Proteins Can Be Transported via the Tat Pathway

**Fig. 2:**
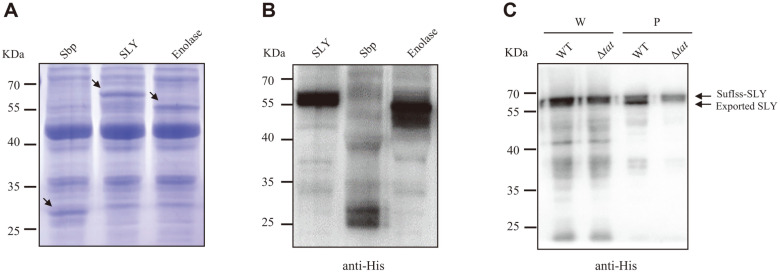
Expression and secretion of *S. suis* antigen proteins. (**A**-**B**) *E. coli* BL21(DE3) containing pQE80-SufIss- SLYhis, pQE80-SufIss-Enohis, and pQE80-SufIss-Sbphis, respectively, was subcultured overnight in LB. IPTG was added with a final concentration of 1 mM when the absorbance at OD_600_ reached 0.3, and the cells were cultured for another 3 h for protein expression induction. The cells were pelleted and the protein expression was analyzed by SDS-PAGE (**A**) and Western blot using anti-His antibody (**B**). (**C**) Cells of *E. coli* MC4100 (WT) and *E. coli* MC4100 Δ**tat** strain (Δ**tat**) containing pJ23-SufIss- SLYhis plasmid were subcultured 1:100 in LB overnight and incubated at 37°C until the absorbance at OD_600_ reached 1. 5 ml and each culture was pelleted by centrifugation. The cell pellet was resuspended with 250 μl of resuspension buffer (50 mM Tris- HCl, pH 7.6, 2 mM EDTA), which was the whole-cell sample (W). Cells were pelleted from 25 ml of each culture and resuspended with 500 μl of fractionation buffer (20 mM Tris-HCl, pH 7.6, 2 mM EDTA, 20% sucrose). Lysozyme (final concentration 0.6 mg/ml) was added, and the supernatant was collected after 20 min incubation at room temperature, which was the periplasmic fraction (P). The whole-cell samples and the periplasmic fraction samples were analyzed by SDS-PAGE followed by Western blotting using anti-His antibody.

We next investigated whether *S. suis* antigen proteins could be transported to the periplasm via the Tat system when fused with a SufI signal peptide. We first showed that SufI signal peptide-fused *S. suis* immunoprotective proteins SLY, Sbp, and Enolase could be successfully expressed in the soluble fraction of *E. coli* MC4100 (Figs. 2A and 2B). To check whether the signal peptide-containing antigen proteins could be exported by the Tat pathway, the proteins were expressed in the WT strain and the Tat system-deficient strain (*E. coli* MC4100 Δ*tatABC*), respectively. Whole-cell samples and periplasmic fractions were prepared and analyzed by Western blot. It was shown in Fig. 2C that a smaller band corresponding to the mature form of SLY could be seen in the WT strain, but not in the Δ*tatABC* strain, indicating that SLY could be exported to the periplasm through the Tat system. These results suggest that *S. suis* antigen proteins, at least SLY, could be exported in a Tat-dependent manner.

### Secretion of the *S. suis* Antigen Proteins to the Extracellular Environment

**Fig. 3:**
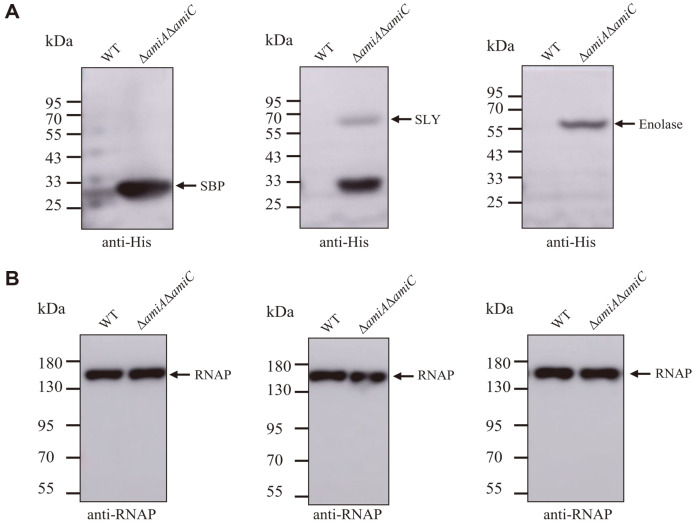
Secretion of the *S. suis* antigens by the outer membrane-defective *E. coli*. *E. coli* Δ*amiA*Δ*amiC* strain containing pJ23-SufIss-SLYhis, pJ23-SufIss-Enohis, and pJ23-SufIss-Sbphis, respectively, was grown overnight in LB with shaking. The same amount of the culture supernatant was collected and concentrated by ultracentrifugation. The samples were then analyzed by SDS-PAGE followed by Western blotting using anti-His antibody (**A**) and anti-RNA polymerase (RNAP) antibody (**B**).

Given that foreign proteins containing a SufI signal peptide can be transported to the periplasm via the Tat system, we next tested whether the *S. suis* antigen proteins that were exported to the periplasm could be further released to the extracellular environment by using an outer membrane-defective strain. N-terminally SufI signal peptide-fused SLY, Sbp, and Enolase of *S. suis* were individually expressed from pJ23 plasmid in the WT and the Δ*amiA*Δ*amiC* strain, respectively. As shown in Fig. 3A, a significantly higher amount of the proteins could be detected in the culture supernatant of the Δ*amiA*Δ*amiC* strain than that of the WT strain, while a comparable amount of RNA polymerase was detected in the culture supernatant of both strains. These results suggest that by exploiting the Tat pathway, the *S. suis* antigen proteins can be successfully secreted to the extracellular environment by using the outer membrane-defective *E. coli*.

### Mice Vaccinated with the *E. coli* Secreting the Antigen Proteins Showed Higher Survival and Milder Clinical Symptoms after the *S. suis* Challenge

**Fig. 4:**
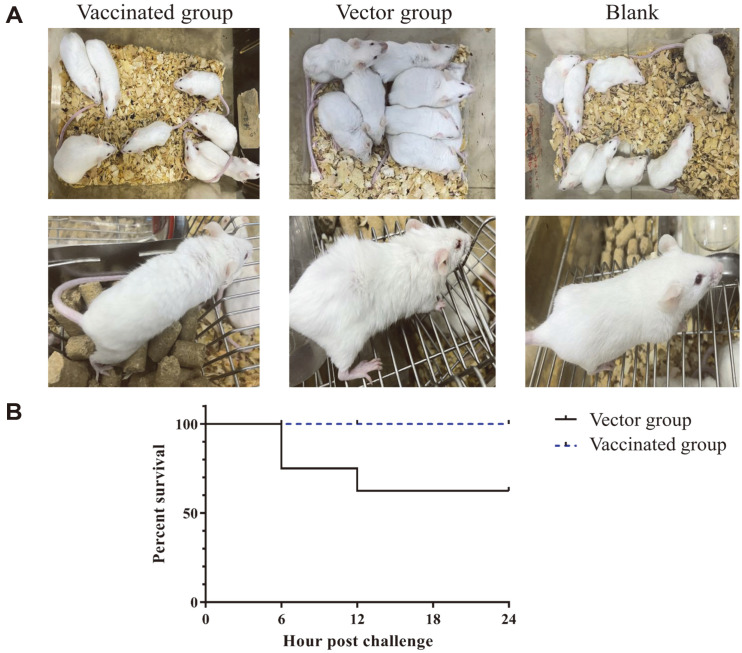
Clinical symptoms and survival. Four-to-five-week-old Kunming mice were randomly divided into 3 groups with 8 mice in each group. The mice of the vaccinated group were inoculated twice with the vaccine by oral gavage. The vector group was administered with the same amount of bacterial cells of the vector stain. The blank group was neither inoculated nor challenged. Seven days after the second vaccination, the mice in the vaccinated group and the vector group were challenged by intraperitoneal injection with 1.5 × 10^9^ CFU of *S. suis* SC19. The clinical symptoms (**A**) and survival (**B**) were recorded.

Based on the above results, we constructed three Δ*amiA*Δ*amiC* strains which constitutively expressed SufI signal peptide-fused antigen proteins SLY, Enolase, and Sbp, respectively, from a plasmid pJ23 containing the constitutive promoter J23119, which were used to inoculate mice. The mice of the vaccinated and vector groups were challenged one week after the second vaccination with *S. suis* SC19, which is a virulent clinical isolate [31]. As shown in Fig. 4A, the vector group mice showed obvious clinical signs, including clustering, depression, shortness of breath, ragged dorsal hair, and copious eyelid discharge after challenge, while the mice in the immunized group were in good mental condition and only showed slightly ragged dorsal hair. The survival rate at 24 h after the challenge was only 62.5% (5/8) for the vector group, while no mice died in the immunized group (Fig. 4B). These results showed that vaccination with the antigen-secreting *E. coli* can provide immune protection against *S. suis* challenge.

### Lower Bacteria Load and Milder Pathological Changes Were Observed in the Organs of Vaccinated Mice

**Fig. 5:**
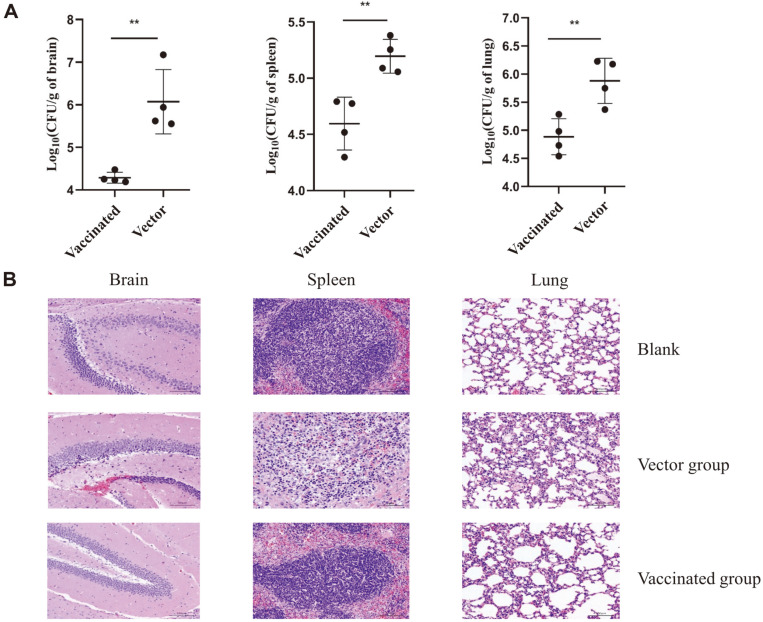
Analysis of bacteria burdens and pathological changes. (**A**) At 24 h after the challenge, 4 mice in each group were euthanized and the organs were collected which were homogenized, diluted with PBS, and plated on TSA plate containing 5% fetal bovine serum for bacteria enumeration. (**B**) 3 mice in each group were dissected 24 h after the challenge, and the brain, spleen, and lung tissues of the mice in each group were collected, fixed in 4% paraformaldehyde, and embedded in paraffin. The samples were cut into thin sections, stained with haematoxylin/eosin (H & E), and examined under a microscope.

To further assess the immune protection efficacy, we analyzed the bacteria loads as well as pathological injury in mice organs including the brain, spleen, and lung after the *S. suis* challenge. As shown in Fig. 5A, the bacteria loads were significantly lower in the organs of the vaccinated group than those of the vector group. In addition, three mice in each group were dissected 24 h after the challenge and the organs were subjected to histopathological examination. As shown in Fig. 5B, the mice in the vector group showed much more severe pathological changes compared to the immunized mice, including large areas of edema and hemorrhage in brain tissues, collapsed alveoli, and thickened alveolar walls, with lymphocyte necrosis, neutrophilia, and hemorrhage in the spleen.

## Discussion

Live vector vaccines have the advantages of low cost, flexibility, and enhanced induction of immune response [1-3]. The most commonly used bacterial vectors to deliver antigens include *E. coli*, *Salmonella*, and lactic acid bacteria as well as virulence-attenuated pathogen bacteria [2, 32-34]. *Salmonella* can elicit an efficient host immune response as it can invade the M cells of the intestine and present antigens effectively [35, 36]. However, in food-producing animals including pigs and poultry, where control and prevention of *Salmonella* is of urgency, using live *Salmonella* as vaccine vectors comes with difficulties in differentiating *Salmonella* infection and *Salmonella* vaccine. *E. coli*, which is frequently used for heterologous protein expression, has also been exploited as a vector for antigen protein delivery [33, 37]. Due to the presence of the cell wall, including the inner membrane and the outer membrane in gram-negative bacteria, the cytoplasmically expressed proteins are difficult to be secreted or exposed at the surface of the bacteria. Therefore, protein secretion systems have been exploited to present antigens on the bacterial surface or for antigen secretion. Byrd *et al*. constructed an attenuated *E. coli* strain secreting Shiga toxin B subunit via the type V secretion system and showed that this strain provided robust immune protection against challenge with enterohemorrhagic *E. coli* (EHEC) [38]. The type III secretion system has also been used to deliver antigens that can be directly injected into the host cells. *Salmonella* delivering PcrV protein through the type III secretion system was used as a vaccine that was shown to be able to provide protection against *Pseudomonas aeruginosa* [39].

In our study, we constructed a live vector vaccine using an engineered commensal *E. coli* strain. To achieve protein export from the cytoplasm, the Tat pathway was exploited. The Tat pathway is unique in that it can transport fully folded proteins, therefore enabling the export of proteins with correct conformation [40, 41]. Targeting heterologous proteins to the Tat pathway can be easily accomplished by fusing a Tat signal peptide to the N-terminus of the protein of interest. We used a SufI signal peptide in which SufI is a well-studied Tat substrate without the need for chaperone proteins for its export [42, 43]. Our results showed that SufI signal peptide can effectively guide the *S. suis* antigen protein to be exported by the Tat pathway (Figs. 1 and 2). Effective targeting of foreign antigen proteins to the Tat pathway by SufI signal peptide has also been reported previously [21].

Another barrier for foreign proteins to be secreted to the extracellular environment is the outer membrane of E. coli. It has been reported that antigen proteins present in the periplasm can not elicit robust immune responses [38]. Thus, we constructed an *E. coli* Δ*amiA*Δ*amiC* strain which has been previously shown to be defective in the outer membrane [30]. Our results showed that secretion of the SufI signal peptide-fused *S. suis* antigen proteins was significantly enhanced by such an *E. coli* strain (Fig. 3). In a previous study, it was shown that high-level secretion of a Tat signal peptide-fused foreign protein to the culture medium was feasible by using a *Bacillus subtilis* Tat system in *E. coli* [20]. In the study, the secretion of foreign protein was achieved in an *E. coli* strain in which the native Tat system was absent. The possible mechanism could be that the outer membrane was leaky in the absence of the *E. coli* Tat system. In our study, the native *E. coli* Tat system is still present and able to export its substrate proteins, and at the same time, deletion of the Tat-dependent amidases leads to a leaky outer membrane, which enhances the release of periplasmic proteins to the extracellular environment. Therefore, our study provides a simple method for protein secretion in *E. coli*.

*S. suis*, which has worldwide distribution, is an important bacterial pathogen threatening the pig industry as well as public health [22, 23]. The most commonly used vaccine in controlling *S. suis* infection in pigs consists of bacterins. However, due to the high diversity in serotypes and sequence types, it is difficult to use bacterins to provide cross-protection [22]. To overcome this problem, subunit vaccines have been studied [44, 45]. Antigen proteins, including SLY [46], Enolase [47], and Sbp [48], have been revealed to provide immune protection against *S. suis* infection. However, so far, no live vector vaccine against *S. suis* has been reported. In this study, we reported that by using the outer membrane-defective *E. coli*, the *S. suis* antigen proteins SLY, Enolase, and Sbp can be effectively secreted via the Tat pathway. This live vector vaccine showed immune protection against *S. suis* infection in a mice model. It should be pointed out that although the vaccine showed protection against *S. suis* infection, the protective efficacy is not very high as the vaccinated group still showed mild clinical signs and bacterial colonization, indicating that further optimizations are needed. Also, by using live engineered bacteria as vaccine, risks including lateral transfer of genes and environmental contamination should be considered. In addition, we use a plasmid to constitutively express the antigen proteins in which plasmid stability and antibiotics resistance concerns need to be considered. Integrating the antigen expression cassette into the chromosome may be applied to solve this problem.

## Supplemental Materials

Supplementary data for this paper are available on-line only at http://jmb.or.kr.

## Figures and Tables

**Table 1:** Bacterial strains and plasmids used in this study.

Strain/plasmid	Description	Source
Strains		
*E. coli* MC4100	A commensal *E. coli* K-12 strain, wild type	[26]
*E. coli* MC4100 Δ*amiA*Δ*amiC*	As *E. coli* MC4100, *amiA* and *amiC* deleted	This study
*E. coli* MC4100 Δ*tatABC*	As *E. coli* MC4100, *tatABC* deleted	This study
Plasmids		
pgRNA-bacteria	Plasmid for sgRNA expression, Amp^r^	[27]
pREDCas9	Plasmid for constitutive expression of Cas9 and inducible expression of the λ Red recombineering system, Spc^r^	[28]
pgRNA-tatABC	As pgRNA-bacteria, expressing the sgRNA targeting the *tatABC* operon. Used in deleting *tatABC*.	This study
pgRNA-amiA	As pgRNA-bacteria, expressing the sgRNA targeting *amiA*. Used in deleting *amiA*.	This study
pgRNA-amiC	As pgRNA-bacteria, expressing the sgRNA targeting *amiC*. Used in deleting *amiC*.	This study
pQE80-SufIss-GFPhis	As pQE80, a DNA fragment encoding GFP with a SufI signal peptide fused to its N-terminus and a C-terminal hexahistidine tag inserted in the MCS, Amp^r^	This study
pQE-SufIss-SLYhis	As pQE80, a DNA fragment encoding *S. suis* SLY with a SufI signal peptide fused to its N-terminus and a C-terminal hexahistidine tag inserted in the MCS, Amp^r^	This study
pQE-SufIss-Enohis	As pQE80, a DNA fragment encoding *S. suis* enolase with a SufI signal peptide fused to its N-terminus and a C-terminal hexahistidine tag inserted in the MCS, Amp^r^	This study
pQE-SufIss-Sbphis	As pQE80, a DNA fragment encoding *S. suis* Sbp with a SufI signal peptide fused to its N-terminus and a C-terminal hexahistidine tag inserted in the MCS, Amp^r^	This study
pJ23	As pQE80, the promoter was replaced with a constitutive J23119 promoter	This study
pJ23-SufIss-SLYhis	As pQE-SufIss-SLYhis, the promoter was replaced with a constitutive J23119 promoter	This study
pJ23-SufIss-Enohis	As pQE-SufIss-Enohis, the promoter was replaced with a constitutive J23119 promoter	This study
pJ23-SufIss-Sbphis	As pQE-SufIss-Sbphis, the promoter was replaced with a constitutive J23119 promoter	This study
