# Supplementary material for: An Engineered Outer Membrane-Defective Escherichia coli Secreting Protective Antigens against Streptococcus suis via the Twin-Arginine Translocation Pathway as a Vaccine
Source: J Microbiol Biotechnol. 2022 Feb 11;32(3):278–86. doi: 10.4014/jmb.2107.07052 (PMC9628857; doi:10.4014/jmb.2107.07052)
Supplement: Supplementary file 1 [file jmb-32-3-278-supple.pdf]

J. Microbiol. Biotechnol.
https://doi.org/10.4014/jmb.2107.07052

Supplementary Table

Table S1. Primers used in this study

Primer	Sequence
pgRNA-upF	CCCCGAAAAGTGCCACCT
pgRNA-downR	TCAATGATGATGATGATGATG
sgRNA-amiA1	TTGGCTGCCCTGACGTTGTCGTTTTAGAGCTAGA AATAGCAAG
sgRNA-amiA2	GACAACGTCAGGGCAGCCAAACTAGTATTATACC TAGGACTGAG
sgRNA-amiC1	GCAAGGCGCGGGTGCCATGTGTTTTAGAGCTAG AAATAGCAAG
sgRNA-amiC2	ACATGGCACCCGCGCCTTGCACTAGTATTATACCT AGGACTGAG
sgRNA-tat1	CGATAATGCAGTTCAGCAGAACTAGTATTATACCT AGGACT
sgRNA-tat2	TCTGCTGAACTGCATTATCGGTTTTAGAGCTAGA AATAGCA
GFP-8001	ACAGAATTCATTAAAGAGGAGAAATTAACTATGA GCCTGAGCCGTCGTC
GFP-8002	CGCAAGCTT TTTGTAGAGCTC
GFP-0101	ATTCCCAATTAAAGGAGGAAGGATCCATGAACAA

	TAACGATCTCTTTC
GFP-0102	AGGCGGGCTGCCCCGGGGACGTCTTAGTGATGG TGATGGTGATGTTTGTAGATCTCATCCATGC
SLY-8001	CAGGTCAGCAGCAGCCGCTGGGATCCGCAGATT CCAAACAAGATATTAATC
SLY-8002	TGGGCCGCAAGCTTTTTGTACTCTATCACCTCATC CGC
Eno-8001	CAGGTCAGCAGCAGCCGCTGGGATCCTCAATTAT TACTGATGTTTACGC
Eno-8002	TGGGCCGCAAGCTTTTTGTATTTTTTCAAGTTGTA GAATGAGTTC
Sbp-8001	CAGGTCAGCAGCAGCCGCTGGGATCCGGTACAT CGAATAGTACAGAC
Sbp-8002	TGGGCCGCAAGCTTTTTGTACTTAGCTTTTGATAC GTCTTC
